# Retrieving transient conformational molecular structure information from inner-shell photoionization of laser-aligned molecules

**DOI:** 10.1038/srep23655

**Published:** 2016-03-30

**Authors:** Xu Wang, Anh-Thu Le, Chao Yu, R. R. Lucchese, C. D. Lin

**Affiliations:** 1 Department of Physics, Kansas State University, Manhattan, KS 66506, USA; 2 Department of Applied Physics, Nanjing University of Science and Technology, Nanjing, Jiangsu 210094, PR China; 3 Department of Chemistry, Texas A&M University, College Station, Texas 77843, USA

## Abstract

We discuss a scheme to retrieve transient conformational molecular structure information using photoelectron angular distributions (PADs) that have averaged over partial alignments of isolated molecules. The photoelectron is pulled out from a localized inner-shell molecular orbital by an X-ray photon. We show that a transient change in the atomic positions from their equilibrium will lead to a sensitive change in the alignment-averaged PADs, which can be measured and used to retrieve the former. Exploiting the experimental convenience of changing the photon polarization direction, we show that it is advantageous to use PADs obtained from multiple photon polarization directions. A simple single-scattering model is proposed and benchmarked to describe the photoionization process and to do the retrieval using a multiple-parameter fitting method.

One of the most important goals of ultrafast atomic, molecular, and optical physics is to observe dynamic structure changes of isolated molecules, in particular, the change of atomic positions versus time so that a molecular movie can be created. Several methods have been proposed or demonstrated, including ultrafast X-ray diffraction (UXD)^1^,^2^,^3^ using the recently developed femtosecond free-electron X-ray lasers, ultrafast electron diffraction (UED)^4^,^5^,^6^,^7^ using short pulses of electron beams, and laser-induced electron diffraction (LIED)^8^,^9^,^10^ using recollision electrons that have tunneled out previously from the target.

Another proposed method, also taking advantage of the femtosecond free-electron X-ray lasers, is ultrafast photoelectron diffraction (UPED)^11^,^12^,^13^. The idea is simple: An electron can be pulled out from an inner-shell orbital that is localized to an emitter atom by absorbing an X-ray photon, and on its way out to the detector the electron can be scattered by the surrounding atoms. The detected electron diffraction pattern is the result of the interference between the direct, or unscattered, electron wave and the scattered electron wave, therefore it encodes molecular structure information, or the transient structure of the molecule in a typical pump-probe scheme after some dynamic structure change has been initiated.

Comparing to the UXD method, which uses X-ray scattering directly, the UPED method needs to convert an X-ray photon into a photoelectron. Doing so gives the following advantages: First, a lower X-ray energy is needed to achieve the same de Broglie wavelength. For example, to achieve the same 1 *Å* de Broglie wavelength, an X-ray photon needs to have an energy of 12 keV whereas an electron only needs to have an energy of 150 eV. The X-ray photon energy needed in the UPED method is thus 150 eV plus the ionization energy of the electron of a specific atomic inner orbital in the molecule, which is about 310 eV for the K shell of carbon and 2.8 keV for the K shell of chlorine, for example. Second, the cross section for electron diffraction is about six orders of magnitude higher than that for X-ray diffraction, so electron diffraction is generally more favorable. Therefore the UPED method has the promise to circumvent the main difficulty for the UXD method of obtaining enough high-energy X-ray fluxes.

Existing proposals on UPED assume that isolated molecules are either fixed in space^11^,^12^ or perfectly aligned along an axis^13^, therefore a molecular-frame or a recoil-frame PAD can be obtained. Molecular structure information can either be decoded by a Fourier transform method^11^,^12^,^14^,^15^ or by a fitting method^13^. Because it is impossible to really fix gas molecules in space or to align them perfectly along an axis, these proposals must rely on the photoelectron-photoion coincidence technique, which uses the asymptotic momentum vectors of the molecular fragments to reconstruct the spatial orientation of the molecule, or of a molecular axis, at the time of ionization (breakup). The limitations of the coincidence technique are also well known. The most severe one is that the count rate must be set very low to avoid false coincidence, and this requirement leads to long data-acquisition time or compromised data quality for limited data-acquisition time, especially at large facilities. Besides, it is not always possible to find a suitable recoil axis, limiting the applicability of this technique.

An alternative approach without using the coincidence technique is to align the gas molecules in space before the X-ray probe pulse. Although perfect alignment cannot be achieved, this approach does not require a low count rate. Each X-ray probe pulse is allowed to ionize many molecules at the same time. Therefore this approach overcomes the main disadvantage of the coincidence technique explained above. The new questions to be answered now are: Can molecular structure information survive a partial-alignment averaging? Or, is it still possible to decode molecular structure information from a PAD that has averaged over a partially aligned ensemble of molecules? If yes, how?

A few experiments along this line have been published recently^16^,^17^,^18^. In refs 16 and 17 the photoelectron energies are limited to a few tens eV and molecular structure retrieval was not attempted. In ref. 18 a higher photoelectron energy (~140 eV) is used and an attempt has been made to retrieve the bond length of a diatomic molecule.

It is therefore desirable and timely to have a comprehensive theoretical study of UPED for laser-aligned gas molecules. It is desirable to know to what degree of alignment molecular structure information can survive. It is desirable to have a theoretical toolkit to decode the available structure information. And it is desirable to be able to retrieve multiple structure parameters at the same time. These are the motivations of this article.

The details of our approach will be explained in the following section but a general idea is given here. We show that even after averaging over partial alignments, the PADs are reasonably sensitive to molecular structure changes. A particular change in the molecular structure leaves a noticeable and unique trace in the alignment-averaged PADs, making structure retrieval possible.

The retrieval process is carried out using a multiple-parameter fitting method: Given an “experimental” PAD corresponding to an unknown transient molecular structure, we use a simple yet accurate photoelectron scattering model containing the molecular structure parameters to simulate a PAD, which has averaged over the alignment distribution, and compare it to the experimental one. If the comparison is not satisfactory, the molecular structure parameters will be adjusted and a new PAD is simulated and compared again to the experimental one. This process is repeated many times until a satisfactory and converged comparison is obtained. The corresponding molecular structure parameters used in the model are the retrieved molecular structure parameters.

The necessity of a simple and accurate model to describe the photoelectron diffraction process and to simulate alignment-averaged PADs is thus obvious. Although more sophisticated molecular photoionization codes are available, such as ePolyScat^19^,^20^, EDAC^21^, CMS-X*α*^22^, to name a few, they are too computationally demanding to be repeated a large number of times for the purpose of fitting. Fortunately, the physical process of inner-shell molecular photoionization is relatively simple and clear, provided that the photoelectron energy is higher than a few hundred eV. Then the photoelectron has a localized origin and the scattering is mainly due to the surrounding atomic centers instead of the delocalized valence electron clouds. This enables us to construct a simple model that is accurate and fast enough to do the retrieval.

## Model and Method

### The single-scattering model

The model that we use in this paper is similar in idea to the single-scattering model used by Krasniqi *et al.*^11^, but here we treat the electron elastic scattering factor more elaborately so that the model agrees quantitatively well with the more sophisticated ePolyScat code^19^,^20^, which is a well established quantum chemistry code designed for electron-molecule scattering and molecular photoionization processes.

**Figure 1:**
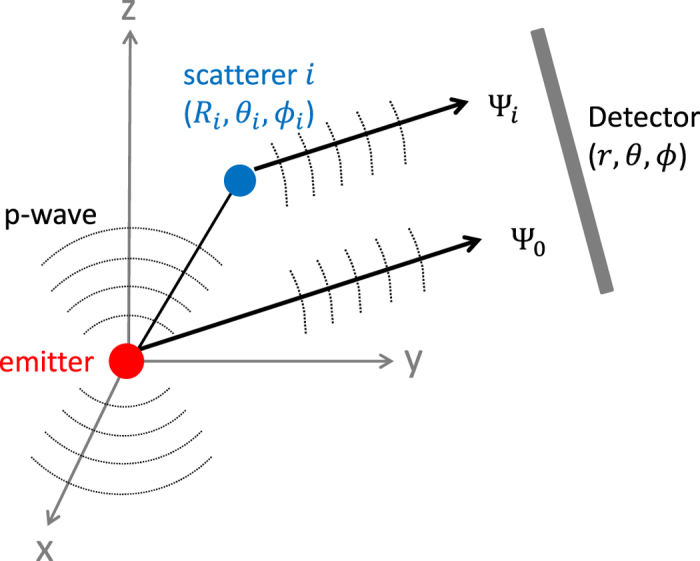
Schematic illustration of photoelectron diffraction. An outgoing p-wave is emitted from the emitter atom and this wave can either go directly to the detector (Ψ_0_) or be scattered by another atom scatterer *i* before reaching the detector (Ψ_*i*_).

A schematic illustration of photoelectron diffraction is given in Fig. 1. An electron is assumed to be pulled out from a localized inner-shell orbital by an X-ray photon. The photoelectron is described by an outgoing spherical wave, the angular distribution of which is determined by the angular momentum of the ionizing orbital: for an s-orbital, the outgoing wave is a p-wave; and for a p-orbital, the outgoing wave is a superposition of an s-wave and a d-wave. In this paper, for the purpose of simplicity in demonstration, we assume the ionizing orbital is an s-orbital, so the outgoing wave is a p-wave. The direction of the p-wave is along the photon polarization direction and it can be written as


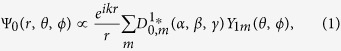


where 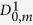 is a Wigner D-function and the summation is over *m* = −1, 0, 1. The direction of photon polarization is controlled by the Euler angles (*α*, *β*, *γ* = 0).

This direct wave can be scattered by the surrounding atoms of the molecule. The elastically scattered wave from atom *i* at position 
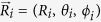
 is


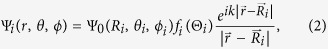


where the first factor on the right hand side is the direct wave at position 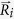, the second factor is the electron elastic scattering factor for atom *i* with Θ_*i*_ the scattering angle, and the third factor denotes an outgoing spherical wave centered at atom *i*.

The detected photoelectron signal at position 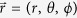 is


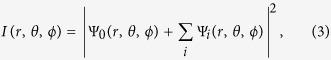


where the summation includes scattered waves from all surrounding atoms.

### The muffin-tin model potential and the electron scattering factor

The electron scattering factor *f*(Θ) is a key quantity determining whether the simple single-scattering model introduced above can provide quantitative agreement with experimental data, or in this paper simulation results from the ePolyScat code^19^,^20^. We found that for photoelectron energies of a few hundred eV, it is not adequate to use the Born approximation to obtain the electron scattering factor, as did by Krasniqi *et al.* in ref. 11, and a more elaborate treatment is needed.

The electron scattering factor is directly determined by the potential that the photoelectron feels during its way out to the detector. This potential is in principle very complicated since it involves the clouds of all the remaining electrons, as well as the nuclei. A widely used technique that has been shown to provide quantitative agreements to experimental photoionization data is to construct a muffin-tin model potential^13^,^21^,^22^,^23^,^24^. The electron scattering factor for each atom scatterer can be obtained after the muffin-tin potential has been constructed.

**Figure 2:**
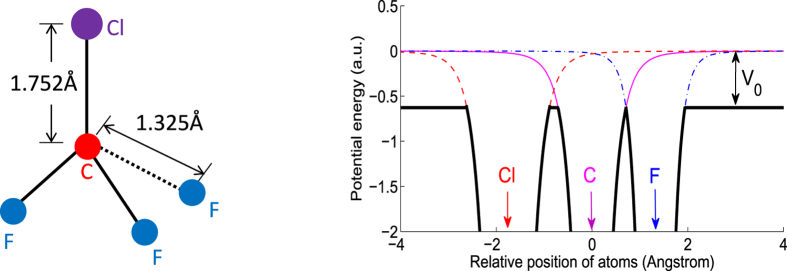
(Left) Experimental equilibrium geometry of the CF_3_Cl molecule. The angle ClCF is 110.3°. (Right) Construction of the molecular muffin-tin potential from free-atom potentials for photoelectron energy 500 eV. The red dashed curve shows the free-atom potential for Cl, the magenta solid curve shows that for C, and the blue dash-dotted curve shows that for F. The free-atom potentials for Cl and F have been shifted in position with respect to C according the experimental Cl-C and C-F bond lengths. The constructed muffin-tin molecular potential is shown as the thick black curves. *V*_0_ is the muffin-tin zero relative to vacuum.

The procedure to construct a model muffin-tin potential is described as follows, using the CF_3_Cl molecule as an example here, the structure of which at equilibrium geometry is shown in the left panel of Fig. 2 25.

First, a free-atom potential is obtained for each atom. To obtain the free-atom potential, an electron density distribution is first calculated using a multiconfiguration Dirac-Fock program of Desclaux^26^. The free-atom potential can then be calculated including the electrostatic interaction potential and the exchange potential. The latter uses an expression derived by Furness and McCarthy^27^. The details of the calculation of free-atom potentials have been documented in ref. 28 by Salvat *et al.* and will not be repeated here. The obtained free-atom potentials are shown in the right panel of Fig. 2. The red dashed curve is the free-atom potential for Cl, the magenta solid curve is for C, and the blue dash-dot curve is for F. The potentials for Cl and F have been shifted with respect to C according to the experimental Cl-C and C-F bond lengths, which are 1.752 *Å* and 1.325 *Å*, respectively.

Second, the muffin-tin molecular potential is constructed using a touching-sphere technique^29^,^30^. The free-atom potential of C first intersects that of F at a distance of 0.71 *Å* away from C (or 0.615 *Å* from F) with an energy of −0.627a.u. (−17.07 eV). This determines the muffin-tin radius of C to be 0.71 *Å* and that of F to be 0.615 *Å*, and the muffin-tin zero energy *V*_0_ = 17.07 eV, which connects the photoelectron energy felt by the molecule (*E*_*p*_) and the photoelectron energy measured in vacuum (*ε*_*p*_) via *E*_*p*_ = *ε*_*p*_ + *V*_0_. The muffin-tin zero energy *V*_0_ in turn determines the muffin-tin radius of Cl to be 0.864 *Å*, so the muffin-tin sphere of Cl does not touch that of C, leaving a nearest distance of 0.18 *Å* between the two muffin-tin spheres.

After the muffin-tin radius of an atom is determined, the electron elastic scattering factor *f*(Θ) for that atom will be calculated using the code ELSEPA^28^, which performs partial-wave calculations using the muffin-tin potential of that atom. After the electron scattering factor for each atom has been obtained, the photoelectron signal can be calculated using the single-scattering model introduced in the previous subsection.

### Applicability range of the model

To test the accuracy of the single-scattering model, we first make a comparison of the photoelectron spectra generated by the code ePolyScat^19^,^20^, which intrinsically includes multiple scattering, and by the single-scattering model, for a fix-in-space CF_3_Cl molecule. The molecule, as illustrated in the left panel of Fig. 2, is fixed in space such that the C-Cl axis is along the z direction and the azimuthal angles of the three F atoms are 90°, 210°, and 330°. The X-ray photon polarization direction is along the z direction also. The photoelectron is assumed to be pulled out from the C 1s orbital.

**Figure 3:**
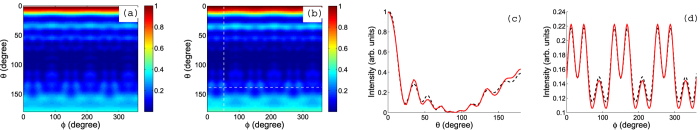
Photoelectron angular distributions calculated (**a**) by the ePolyScat code and (**b**) by the single-scattering model. The photoelectron is pulled out from the C 1s orbital and its energy is 500 eV. Each spectrum is normalized to its own maximum value. The step size is 2.5° for both *θ* and *ϕ*. Two representative slices are selected from each spectrum for a further comparison, as indicated by the two white dashed lines in panel (**b**). (**c**) Is for the vertical slice with *ϕ* = 50° and (**d**) is for the horizontal slice with *θ* = 140°. The black dashed curves are the ePolyScat results and the red solid curves are the model results.

For photoelectron energy 500 eV, Fig. 3 shows the PADs generated by (a) ePolyScat and (b) the single-scattering model. Each spectrum has been normalized to its own maximum value. One can see that the two PADs agree very well visually. We also select two representative slices from each spectrum for a further comparison, a horizontal one (*θ* = 140°) and a vertical one (*ϕ* = 50°), as denoted by the two white dashed lines in panel (b). The comparisons are shown in panels (c) and (d). The black dashed curves are for ePolyScat and the red solid curves are for the model. One can see that for both slice cuts the model agrees quite well with ePolyScat.

**Figure 4:**
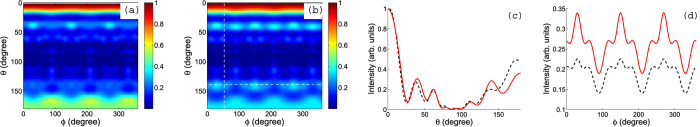
Same asFig. 3, but for photoelectron energy 300 eV.

Similar comparisons have also been made for a lower photoelectron energy of 300 eV, as shown in Fig. 4. Visual differences between the two PADs can be seen from panels (a) and (b), especially for *θ* close to 180°. These differences are attributed to multiple-scattering effects which are included in ePolyScat but not in the single-scattering model. From Figs 3 and 4, we conclude that the single-scattering model works for photoelectron energies about 500 eV and above.

### From fix-in-space to partial-alignment

The PADs shown in Figs 3 and 4 assume the molecule is fixed in space and the photon polarization axis is parallel to the molecular C-Cl axis. In fact, for a symmetric top molecule like CF_3_Cl, it is not possible to align it in 3D. The best that one can do is to align it in 1D along the C-Cl axis.

**Figure 5:**
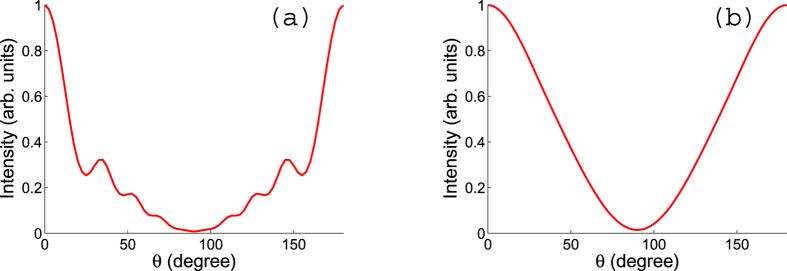
PADs for (**a**) perfectly 1D aligned ensemble of CF_3_Cl molecules and for (**b**) partially 1D aligned ensemble of CF_3_Cl molecules with alignment degree 0.82. The information in *ϕ* is lost so only dependence on *θ* is shown. The photoelectron is from the C 1s orbital with energy 500 eV.

For 1D aligned CF_3_Cl molecules along the C-Cl axis, the absolute azimuthal orientation of the F atoms is random. The resulting PAD must average incoherently over all possible azimuthal angles. This averaging erases information in *ϕ*. The oscillation along *θ* (which is the polar angle from the z axis) remains, however. For example, Fig. 5(a) shows the oscillation in *θ* for an ensemble of perfectly 1D aligned CF_3_Cl molecules. This means that the C-Cl axis is exactly along the z axis (Both up and down are allowed though, otherwise the molecule is called oriented, instead of aligned. Orientation cannot be achieved using an aligning laser alone.)

However, the oscillations along *θ* are quickly washed away, if the molecules are not perfectly aligned, meaning that the C-Cl axis is allowed to wobble about the z axis. The degree of alignment is usually quantified by the average value of 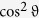: 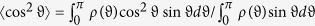, where *ϑ* is the angle between the z axis and the C-Cl axis and *ρ*(*ϑ*) is the distribution function of *ϑ*. For example, if 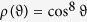 then 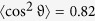. Panel (b) of Fig. 5 shows the PAD with this alignment distribution. We can see that the oscillations in *θ* shown in panels (a) are washed away and the PAD is rather smooth in *θ*.

A brief general theory of PADs would be helpful. For a random ensemble of gas molecules, the PAD has the following form


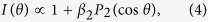


where *θ* is the polar angle from the photon polarization axis, *P*_2_(*x*) = (3*x*^2^ − 1)/2 is the 2nd order Legendre polynomial, and *β*_2_ is called an asymmetry parameter.

For a partially aligned ensemble of gas molecules where the alignment axis is parallel to the photon polarization axis, the above formula is extended to include higher-order Legendre polynomials^31^,^32^


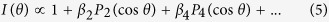


The better the alignment, the more higher-order Legendre polynomials contribute, the more structures the PAD has.

Information about the molecular structure is encoded in the *β* parameters. In principle, even for a random ensemble of molecules without alignment, a different molecular structure will give a different *β*_2_ value. The question is whether this difference is large enough to be measured experimentally. This difference is more substantial if the molecules are aligned, with which more *β* parameters are included.

The goal of this paper is to show that although a PAD becomes much more structureless after averaging over partial alignment distributions, it may still be sensitive enough to a change in the molecular structure, provided that the molecules are moderately aligned. A particular change in the molecular structure leaves a unique and noticeable footprint in the PAD, from which the change in the molecular structure can be retrieved.

### PAD using multiple photon polarization directions: the 2D “mPAD”

**Figure 6:**
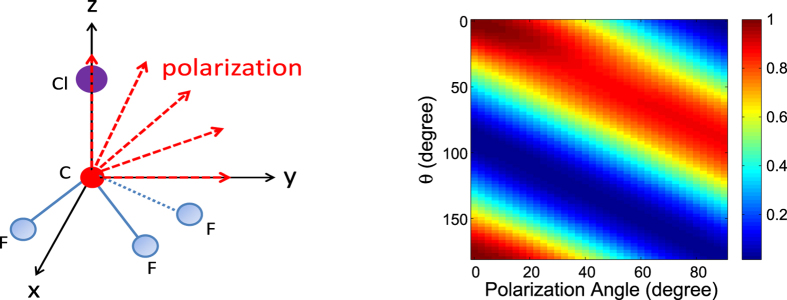
(Left) The CF_3_Cl molecule is laser-aligned along the z axis while multiple polarization directions are used. The polarization directions are assumed to be within the y-z plane with the polarization angle changing from 0° (z axis) to 90° (y axis). (Right) Example of an “mPAD”. Each polarization direction contributes a 1D PAD (as a function of *θ*) and multiple polarization directions lead to a 2D mPAD as demonstrated by this panel. The molecules are assumed to be partially aligned along the z axis with a degree of alignment of 0.82. The photoelectron is from the C 1s orbital with energy 500 eV.

In the previous subsection we have discussed alignment-averaged PAD for a single photon polarization direction, which is assumed to be parallel to the molecular alignment axis. Experimentally it is actually easy to change the relative angle between these two directions. Exploiting this experimental convenience, we consider PADs obtained with multiple polarization directions while keeping the molecular alignment axis fixed. (Or equivalently we can fix the photon polarization direction and use multiple molecular alignment directions.) As illustrated in the left panel of Fig. 6, the molecular alignment axis is assumed to be along the z axis and the photon polarization axis changes continuously from the z axis to the y axis within the y-z plane. For each polarization direction, a 1D PAD as a function of *θ* is collected. Note that *θ* is the polar angle from the z axis, which is the molecular alignment axis here, and that the detection is also within the y-z plane. Multiple polarization directions compose a 2D PAD, as shown in the right panel of Fig. 6. The horizontal axis is the polarization angle, changing from 0° (z axis) to 90° (y axis), and each polarization angle contributes a 1D PAD as a function of *θ*. To distinguish this 2D PAD composed of multiple photon polarization directions from the usual PAD for a single polarization direction, we call the former an “mPAD” for the time being, denoting the use of multiple polarizations.

The use of multiple polarization directions fully exploits the experimental convenience of changing the polarization direction. Although a change in molecular structure will lead to a change in the PAD for any polarization direction, it is expected that the PAD for some polarization direction may be more sensitive to a certain structure parameter than for other polarization directions. This is indeed what we found. This means that if a structure parameter changes, the 2D mPAD shown in Fig. 6 (right) will change nonuniformly for different polarization directions. This is the reason why we use multiple polarization directions and construct the 2D mPAD.

### Change in mPAD when the molecular structure changes

**Figure 7:**
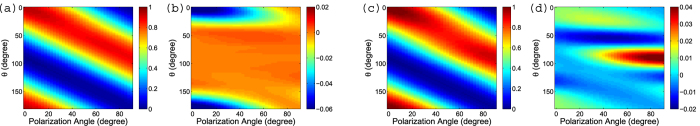
(**a**) New mPAD if the C-Cl bond length increases by 0.1 *Å*; (**b**) The difference between panel (**a**) and Fig. 6 (right), which is the original mPAD for the equilibrium structure; (**c**) mPAD if the Cl-C-F angle decreases by 20°; (**d**) The difference between panel (**c**) and Fig. 6 (right).

Figure 7 shows how the mPAD changes if the structure of the CF_3_Cl molecule changes slightly from its equilibrium. For example, if the C-Cl bond length increases by 0.1 *Å* (about 5.7% of the equilibrium value) while all other structure parameters remain the same, the new mPAD is shown in Fig. 7(a). The difference between the new and the original mPAD at equilibrium [Fig. 6 (right)] is shown in panel (b). One can see that the main difference appears near *θ* = 0° and 180° and for the polarization angle less than about 40°. A noticeable change as large as 6% is found in this region.

For another example, if the Cl-C-F angle (for all the three F atoms) decreases by 20° (from about 110° to 90°) while all other structure parameters remain the same, then the new mPAD is shown in panel (c). The difference from the original one is shown in panel (d). One can see that the region for *θ* around 90° and for the polarization angle greater than about 50° is more sensitive to the change of the Cl-C-F angle. A noticeable change of about 4% is seen in this region.

These two examples illustrate why we propose to use multiple polarization directions and construct the 2D mPAD. Different photon polarization directions are sensitive to different molecular structure parameters. As shown in the above examples, polarization along the y axis (with polarization angle 90°) is very insensitive to the change in C-Cl bond length, therefore this bond length cannot be retrieved if only this polarization direction is used. Also, polarization along the z axis (with polarization angle 0°) is insensitive to the change in the Cl-C-F bond angle, therefore this bond angle may not be reliably retrieved if only this polarization direction is used. By using multiple polarization directions, however, multiple molecular structure parameters may be retrieved at the same time.

### Retrieving the change in molecular structure from the change in mPAD

In a real experiment it is the inverse problem that we are faced with. Consider the following experimental scheme. An original mPAD is first obtained for molecules in equilibrium structure. At time zero the molecules are pumped to initiate some dynamic structure change. Then an X-ray pulse is applied at different time delays to pull out the photoelectron and a new mPAD is obtained for each time delay. We are given the new mPAD (or equivalently the difference between the new and the original) for each time delay, and asked to retrieve the instantaneous molecular structure at these time delays. If we can do this, we build a “molecular movie” showing the temporal evolution of the molecular structure.

Since a change in molecular structure leads to a unique change in the mPAD, the former can be retrieved using a fitting procedure: we adjust the molecular parameters, calculate the corresponding mPAD, and compare with the experimental one to see whether they are close enough. This adjusting and comparing process is repeated until a satisfactory agreement is reached between the theoretical and the experimental mPAD.

In practice how this fitting is performed depends on the size of the parameter space involved. If the parameter space is small, e.g., only one or two parameters are changing, one may just enumerate the possible parameter combinations. If the parameter space is large, then more advanced fitting methods are desirable, for example, a genetic algorithm (GA)^33^.

## Results

**Figure 8:**
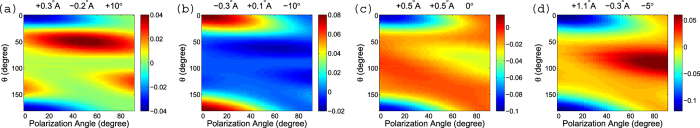
Change in mPAD if three molecular structure parameters change simultaneously. The three structure parameters are the C-Cl bond length, the C-F bond length, and the Cl-C-F bond angle. The change in these three parameters from their equilibrium values are given on top of each panel, in the same order as mentioned. The photoelectron is from the C 1s orbital with energy 500 eV.

In Fig. 7 we showed two examples how the mPAD changes with the change of a single molecular structure parameter. In Fig. 8 we show a few examples how the mPAD changes with a simultaneous change of three molecular structure parameters.

The three parameters are: the C-Cl bond length, the (three) C-F bond length, and the (three) Cl-C-F bond angle. From Fig. 2 (left) the equilibrium experimental values of the three parameters are 1.752 *Å*, 1.325 *Å*, and 110.3°, respectively. We change the three parameters from their equilibrium values and this leads to changes in the mPAD, as shown in Fig. 8 (Raw mPADs are not shown). The label on top of each panel shows how much these three parameters change, for example, (+0.3 *Å*, −0.2 *Å*, +10°) means that the C-Cl bond length increases by 0.3 *Å*, the C-F bond length decreases by 0.2 *Å*, and the Cl-C-F angle increases by 10°.

One can see that each parameter combination leaves a unique and substantial footprint on the mPAD. The first three cases (a–c) change by a range of roughly 10% and the last case (d) changes by a larger range of about 17%, due to a larger change in the C-Cl bond length. These unique and substantial footprints enable us to retrieve the molecular structure parameters.

We use a genetic algorithm^33^ to do the retrieval. Because real experimental data with 500 eV photoelectron energy are not yet available, we use the calculated mPAD as the “experimental data”. Pretending that we do not know the corresponding molecular structure, we retrieve the input molecular structure parameters. For example, given the mPAD in Fig. 8(a) as the experimental data, denoted as *S*_0_(*θ*, *χ*), where *χ* is the polarization angle, we search the three parameters within a parameter space of size (±1 *Å*, ±1 *Å*, ±20°) around the input values. For any given parameter combination, an mPAD *S*(*θ*, *χ*) is calculated and compared with the experimental one. The comparison is quantified by a reliability factor, or R-factor, defined as^13^


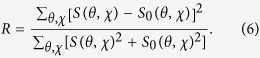


If the comparison is not satisfactory, the parameter combination is adjusted within the parameter space, and a new mPAD is calculated and compared with the experimental one again. This adjusting plus comparing process repeats until the global minimum of R-factor is found.

For three parameters, we find that the correct parameter combination can be easily retrieved using GA. With a population size of five (meaning that each generation has five members), the correct parameter combination can usually be found within a few hundred generations, for all the four cases shown in Fig. 8.

**Figure 9:**
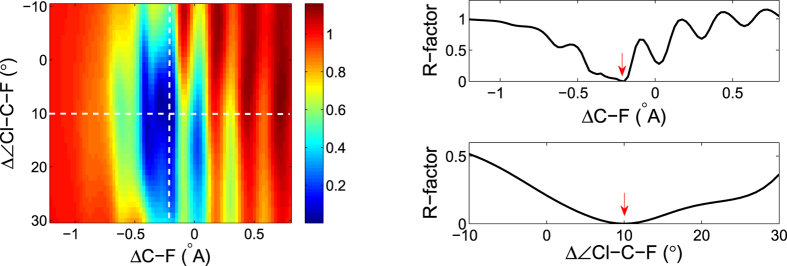
(Left) R-factor image scanning over the C-F bond length and the Cl-C-F bond angle. Bluer region means lower R-factor values thus better fitting, and redder region means larger R-factor values thus worse fitting. The two slice cuts, one horizontal and one vertical, are shown in the right two curves. The red arrows are added by hand to indicate the position of global minimum.

It needs to be pointed out that GA only gives the parameter combination that globally minimizes the R-factor within the parameter space. It does not tell how stable (wide) the global minimum is or whether other local minima exist. To obtain this information, we need to scan over the parameter space and obtain the R-factor value for each point in the parameter space. Taking Fig. 8(a) as an example, the global minimum should be located at the input parameter values (+0.3 *Å*, −0.2 *Å*, +10°). For the sake of easier presentation, in Fig. 9 we show the R-factor map for a sub-parameter space with the C-Cl bond length fixed at the correct value of +0.3 *Å*, then the dependence of the R-factor on the other two parameters, the C-F bond length and the Cl-C-F bond angle, is shown in the color image. The global minimum is located at the center of the image. The C-F bond length covers a 2 *Å* range from −1.2 *Å* to 0.8 *Å* and the Cl-C-F bond angle covers a 40° range from −10° to 30°. On the color image, bluer region means smaller R-factor thus better fitting to the input mPAD; redder region means larger R-factor thus worse fitting to the input mPAD.

Two slice cuts, one horizontal and one vertical as indicated by the two white dashed lines on the image, are also shown on the right panel of Fig. 9. The red arrows indicate the position of the global minimum (−0.2 *Å*, +10°). Scanning along the bond length, one sees a series of local minima in addition to the global minimum and we find that this feature is general, as will be confirmed in a later example. Scanning along the bond angle, the R-factor does not have multiple local minima, but it is quite wide around the global minimum.

**Figure 10:**
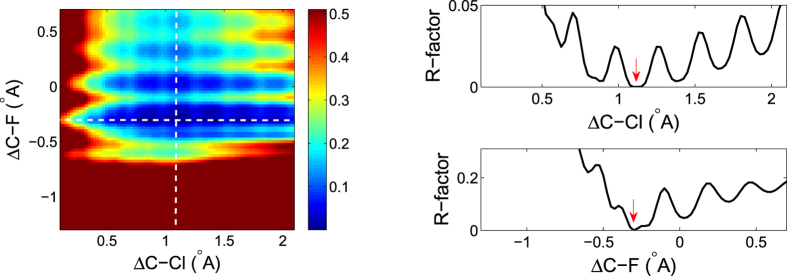
(Left) R-factor image scanning over the C-Cl bond length and the C-F bond length. Bluer region means lower R-factor values thus better fitting, and redder region means larger R-factor values thus worse fitting. The two slice cuts, one horizontal and one vertical, are shown in the right two curves. The red arrows are added by hand to indicate the position of global minimum.

Let us take panel (d) of Fig. 8 for another example. This time we present the R-factor as a function of the C-Cl and C-F bond lengths by keeping the bond angle fixed to the input value (−5°). The 2D R-factor image is shown in the left panel of Fig. 10 with the input values located at the center of the image (1.1 *Å*, −0.3 *Å*). Two slice cuts, one horizontal and one vertical as indicated by the two white dashed lines on the image, are shown in the right panel. The red arrows are inserted to show the position of the global minimum. One can see that similar multiple-minima structures appears when the R-factor scans over each bond length, although the global minimum can be clearly distinguished. In this example the R-factor is more sensitive to the C-F bond length than the C-Cl bond length. This is because the C-Cl bond length (1.752 *Å* + 1.1 *Å* = 2.852 *Å*) is much longer than the C-F bond length (1.325 *Å* − 0.3 *Å* = 1.025 *Å*) and the photoelectron wave amplitude from the emitter C atom is inversely proportional to the bond length.

**Figure 11:**
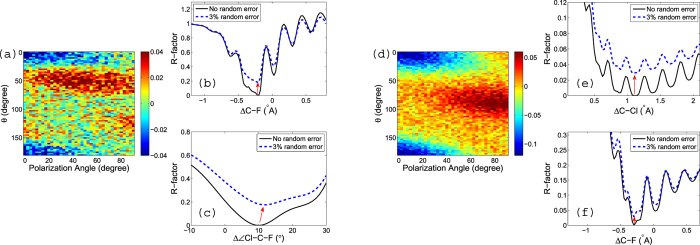
(**a**) Example of a noisy mPAD corresponding to Fig. 8(a). (**b,c**) R-factor curves with and without random errors. The black curves are the same R-factor curves shown in the right panel of Fig. 9; The dashed blue curves show the effect of 3% random errors *in the raw mPADs*. (**d**) Example of a noisy mPAD corresponding to Fig. 8(d). (**e,f**) R-factor curves with and without random errors. The black curves are the same R-factor curves shown in the right panel of Fig. 10; The dashed blue curves show the effect of 3% random errors in the raw mPADs. The red arrows are inserted by hand to indicate the change in the global minimum.

Both examples above show that when the R-factor scans over a bond length, a multiple-minima structure appears. The global minimum, however, can be distinguished. The distinction of the global minimum from the local minima is not surprising, because up to now everything is “ideal” in that the input mPAD image is calculated by the same model that is used for the retrieval. A real experimental mPAD will not be so ideal, for example, it will contain some random errors. Then how robust is the above method if the input mPAD contains random errors? Will random errors lead to a fake retrieved global minimum which is actually one of the local minima? To check this point we assume that *the mPAD at the equilibrium position and at the displaced geometry have some random errors*. We add 3% random errors to each raw mPAD, with respect to its maximum value. We then take the difference between these two mPADs to get a difference mPAD pattern to which the model calculations will fit. Two examples of difference mPADs are shown in Fig. 11(a,d). These two examples correspond to panels (a) and (d) of Fig. 8, where no noises were included.

Now these two noisy mPADs are used as our experimental data and we fit our model calculations to them. The resulting R-factor curves are shown in Fig. 11 (dashed blue curves). One can see that with the inclusion of these random errors, the R-factor at the global minimum is no longer zero as in the ideal case without errors. However, the position of the global minimum does not change substantially. The global minima in (b), (e), and (f) for the bond lengths remain almost exactly the same, and the one in (c) for the bond angle changes for about 1° only. Therefore we can conclude that our method is robust against the inclusion of at least a moderate amount of random errors.

## Discussions on Other Practical Issues

**Figure 12:**
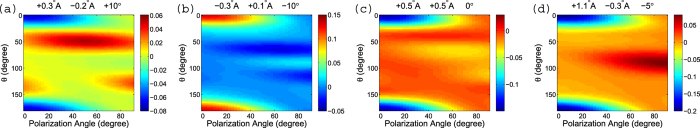
Same asFig. 8, but with a degree of molecular alignment of 0.89.

1. Inclusion of more molecular structure parameters: For the purpose of easier demonstration, we have included three molecular structure parameters for the most part of this paper. This is sufficient for us to deliver our main message that photoelectron diffraction method may be used to retrieve ultrafast changes of multiple molecular structure parameters. There is no fundamental limitation on the number of parameters in our method. More molecular structure parameters can be included and retrieved, provided that the mPAD is “sufficiently sensitive” to this parameter. It is indeed possible that the mPAD is very insensitive to some molecular parameters, especially those associated with a light atom, and those parameters may not be retrieved. Therefore given a target molecule, it would be helpful to use the model to first examine which parameters may be retrieved and which may not.
2. The pump ratio: We have considered a pump-probe scheme that initiates some dynamic molecular structure change. The X-ray pulse is applied at different time delays to generate photoelectron spectra and then the present method is applied to retrieve the change of the molecular structure parameters. In real experiments only a fraction of molecules are pumped, say 10% or 15%. In the fitting process the pump ratio will be an additional parameter in the multi-parameter optimization algorithm.
3. Effect of molecular alignment: The majority of the above PADs assume a degree of molecular alignment of 0.82. This is a moderate degree of alignment that can be achieved in real experiments using laser alignment^16^,^17^. Recently a degree of alignment of 0.97 has been reported with molecules containing heavy atoms^34^. We show in Fig. 12 the change in mPAD with a higher degree of molecular alignment of 0.89 (corresponding to an alignment distribution 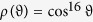), to be compared with Fig. 8 which uses a degree of alignment of 0.82. One can see that the shape of the mPADs is quite similar with a higher degree of alignment except that the amplitude (i.e., the color scale) is larger. This means that the mPADs change more substantially from the equilibrium ones with the molecular structure parameters changing the same amount. A more substantial change in mPAD is likely to be more robust against experimental errors.
4. Number of polarization directions: Experimentally it is easy to change the photon polarization direction with respect to the molecular alignment direction and that is one of the reasons we propose to use multiple polarization directions to construct the 2D mPAD. In real experiments one does not need to use too many polarization directions. A few (say, 5 to 10) are sufficient. We have checked that the retrieved molecular structure parameters have no significant difference by using 5 polarization directions, compared to using 10 polarization directions. This observation can reduce the experimental data collection time, as well as the computational fitting time.

## Conclusion

In this paper we discuss a scheme to retrieve transient molecular structure information using photoelectron angular distributions that have averaged over partial alignment distributions. Ultrafast photoelectron diffraction is a promising candidate to observe dynamic structure change of isolated molecules, along with several other methods such as ultrafast X-ray diffraction, ultrafast electron diffraction, and laser-induced electron diffraction.

We propose to use multiple polarization directions, exploiting the experimental convenience of changing the photon polarization direction. PADs from multiple polarization directions are gathered on a 2D “mPAD”, as we called. A change in molecular structure parameters leaves a unique and noticeable footprint on the 2D mPAD, which enables the retrieval of the molecular structures.

We use a multiple-parameter-fitting procedure to do the retrieval. The fitting procedure requires a fast and accurate model to calculate the alignment-averaged PADs and we have proposed and benchmarked such a model. Our model is a single-scattering model, similar in idea to the one proposed by Krasniqi *et al.*^11^, but with a more elaborate treatment on the electron scattering factors. We have benchmarked our model against the ePolyScat code, which is a well-known quantum chemistry code designed for electron-molecule scattering and molecular photoionization processes^19^,^20^, and found that our simple single-scattering model is quite accurate for photoelectron energies above about 500 eV.

We believe that the idea of retrieving molecular structure information directly from alignment-averaged PADs is practical^18^ and promising in fully realizing the potential of ultrafast photoelectron diffraction.

## Additional Information

**How to cite this article**: Wang, X. *et al.* Retrieving transient conformational molecular structure information from inner-shell photoionization of laser-aligned molecules. *Sci. Rep.* **6**, 23655; doi: 10.1038/srep23655 (2016).
